# Thinking after Drinking: Impaired Hippocampal-Dependent Cognition in Human Alcoholics and Animal Models of Alcohol Dependence

**DOI:** 10.3389/fpsyt.2016.00162

**Published:** 2016-09-30

**Authors:** Miranda C. Staples, Chitra D. Mandyam

**Affiliations:** ^1^Committee on the Neurobiology of Addictive Disorders, The Scripps Research Institute, La Jolla, CA, USA

**Keywords:** alcohol use disorder, cognitive impairment, abstinence, hippocampus, prefrontal cortex

## Abstract

Alcohol use disorder currently affects approximately 18 million Americans, with at least half of these individuals having significant cognitive impairments subsequent to their chronic alcohol use. This is most widely apparent as frontal cortex-dependent cognitive dysfunction, where executive function and decision-making are severely compromised, as well as hippocampus-dependent cognitive dysfunction, where contextual and temporal reasoning are negatively impacted. This review discusses the relevant clinical literature to support the theory that cognitive recovery in tasks dependent on the prefrontal cortex and hippocampus is temporally different across extended periods of abstinence from alcohol. Additional studies from preclinical models are discussed to support clinical findings. Finally, the unique cellular composition of the hippocampus and cognitive impairment dependent on the hippocampus is highlighted in the context of alcohol dependence.

## Occurrence and Impact of Alcohol Use Disorders in the United States

In the United States, 18 million individuals (7.4% of the 15 and older population, according to estimates from 2010) report having an alcohol use disorder (AUD), with nearly 12 million of these individuals reporting alcohol dependence (1). Recent changes to the diagnostic definition of AUDs in the updated DSM-V eliminate the clinical distinction between AUDs and alcohol dependence, opting to categorize them together under the umbrella category of AUDs and describe the broad disorder as a “… problematic pattern of alcohol use leading to clinically significant impairment or distress …” as well as requiring concurrent escalation of alcohol intake, craving for alcohol, and significant disruptions to personal and professional conduct (2). In 2011, AUDs cost the United States $223.5 billion, an estimation which includes the cost of medical treatment, judiciary involvement, and loss of productivity (3).

However, these statistics, while useful in conveying the gravity of the alcohol abuse problem in the United States, do not provide insight into the recovery process nor the continuing health and cognitive disparities these individuals face into periods of abstinence from alcohol consumption. Additionally, long-term alcohol abuse results in significant, non-economic personal costs, including devastating bodily harm, with some of the most striking effects apparent in the brain. Evidence from human and animal studies suggest that select regions of the cortex, particularly the prefrontal cortex (PFC) and hippocampus, may be more sensitive to the deleterious and damaging effects of long-term alcohol use than others, and recovery of cognitive function sensitive to these regions may occur at different times into periods of prolonged abstinence (4–7).

## Impact of Alcohol on Cognition: Clinical Findings

Alcohol is widely known to acutely alter cortical function by modulating inhibitory and excitatory receptor function on neuronal processes (8–10). By repressing excitatory transmission (8, 11–15) and concurrently enhancing inhibitory transmission (16–21), alcohol acutely acts as a systemic depressant. Over repeated, chronic exposures, neuronal transmission achieves a homeostatic state in the presence of alcohol (22), and cognition can resemble that of non-dependent function. However, during periods of abstinence when alcohol is absent from the system for extended phases, effectively disrupting the previously described modified homeostasis, cognitive function is significantly impaired (due to the absence of alcohol as critical modulating factor), and these cognitive impairments persist for some time. Interestingly, these cognitive perturbations, in some instances, do recover to or near pre-dependency levels. What follows is a description and synthesis of how alcohol modulates PFC and hippocampal function, what changes occur as occasional alcohol consumption becomes chronic consumption, and what cognitive impairments are present during acute withdrawal.

It is worth noting, while outside the general scope of this review, that chronic alcohol use does result in structural and/or functional atrophy in regions outside of the PFC and hippocampus and that these additional changes cannot be eliminated as potential modulators of the deleterious effects observed in the PFC and hippocampus (23). Further, research into the cognitive capacities of alcoholic individuals has identified cognitive disorders, such as Wernicke–Korsakoff syndrome, alcohol dementia, and Marchiafava–Bignami disease, which are directly related to long-term alcohol abuse and cloud our understanding of alcohol’s solitary effects on cognitive functioning (24, 25). Similarly, age and concurrent drug use can additionally complicate our understanding of alcohol’s impact; therefore, for the purpose of this review, studies including subjects with chronic alcohol use without poly drug use were evaluated.

## Cognitive Impairment Following Nondependent Alcohol Use

### Prefrontal Cortex

The PFC is a region of the cerebrum, which has been colloquially referenced as the switchboard of the cortex due to its role in planning and selecting appropriate responses and actions to events and stimuli (26–28). Behaviors such as impulsivity (29), decision-making (30), and attentional focus (31) are all under the control of the PFC and are often manipulated and impaired in individuals with an AUD (discussed subsequently). Whenassessed in a controlled setting, acute doses of alcohol (0.4–0.8g/kg) given to nondependent subjects impairs numerous PFC functions, including disruption in planning (32), increases in impulsive actions (33–36), decreases behavioral inhibition (37–39), reduces perseverance (40), and increases poor decision-making (41). In many studies, these dysfunctions were correlated with reductions in typical lateralization (asymmetric distribution of activity) (36) as well as reduced functional magnetic resonance imaging (fMRI) activity during false responses (42). Further, studies in humans have demonstrated subtle structural abnormalities (43), increased blood flow (as an indicator of cortical activity) (44–47), and reduced hemispheric dominance (36, 48–50). Taken together, it is clear that the function of the PFC is significantly impaired with acute exposures to alcohol.

### Hippocampus

Similar to the inhibition observed in the PFC, the hippocampus is a sensitive target of alcohol’s actions in the brain. Defined, in part, by its characteristic trisynaptic circuit, human and animal studies have demonstrated that the hippocampus is critical for spatial memory [reviewed in Ref. (51)], context discrimination (52), pattern separation (53), and time-sensitive memories (54). A critically unique region of the hippocampus, the dentate gyrus (DG), contains neural stem cells that continue to divide and primarily generate functional neurons into adulthood in nearly all mammalian species (55) and have proved critical for pattern separation functionality (56). Beyond its role in the previously described functions, the hippocampus plays a critical role in emotional and stress regulation (57), critical components to the development and cyclical nature of addiction (58). In human subjects, hippocampal function is typically assessed as contextual memory or episodic memory, both of which have been shown to be impacted during acute alcohol exposure (49, 59).

## Cognitive Impairments During and Following Heavy Alcohol Use

### Prefrontal Cortex

When compared with healthy subjects, individuals reporting chronic alcohol abuse demonstrate structural abnormalities, including reduced frontal cortical volume (60–64), compromised white matter integrity (65–67), reduced quantities of frontal–cerebellar connections (68), and aberrant patterns of frontal cortical activity (69, 70). Further, Kril et al. (71) confirmed previously reported reductions in PFC white matter and found a significant reduction in the number of neurons in postmortem tissue of alcoholics when compared with healthy control subjects, confirming losses to cortical gray matter (60). Finally, it is possible that these pathological changes are underlying the diminished cognitive function often observed in human alcoholics.

In order to test the deleterious effects of chronic alcohol abuse on the intellectual capacities of alcohol-dependent individuals, tests of memory, impulsivity, risk, and attention are often employed. While individuals struggling with alcohol dependence rarely exhibit impairments on assessments of generalized intelligence, specialized complex tasks are uniquely able to elucidate potentially subtle difference between dependent and non-dependent populations. Estimates suggest that at least half of individuals diagnosed as alcohol-dependent are also cognitively challenged (4). One early study assessing a group of recently abstinent alcoholics, individuals with frontal lobe damage, and healthy controls found, as expected, no difference on assessments of IQ, but did report that alcoholic individuals were significantly impaired compared with both controls and individuals suffering from frontal lobe trauma in tasks that were designed to explicitly test frontal lobe function (72, 73). More recent studies have demonstrated explicit impairments on tasks, involving executive functioning (74, 75), working memory (76, 77), and impulsivity (76, 78–81). Structural abnormalities have been directly linked to frontal cortical function in within-subject experimental designs. One study measuring frontal cortical electrical activity (electroencephalogram recordings) during a Go/No Go task, a test where subjects are asked to learn and perseverate changing rules pertaining to cues, demonstrated blunted activity during the task in alcoholics as compared with non-dependent controls (82). Most recently, Nakamura-Palacios et al. (83) reported that the damage to the PFC was predictive of the cognitive impairments on tests of executive function. Additionally, studies have identified abnormal patterns of activity during cognitive tasks in alcohol-dependent subjects, whose intellectual performance is comparable to non-dependent subjects (84); this finding is particularly intriguing as it implies that individuals with significant disruptions in cognitive capacities may lack the capacity to form adaptive connections in the presence of chronic alcohol. Taken together, these findings present solid evidence that the PFC is subject to extensive damage as a result of chronic alcohol use, some of which could potentially be mediated by certain individual characteristics.

### Hippocampus

Studies involving human subjects with chronic alcohol use have demonstrated reduced hippocampal volume (85–87), postmortem evidence of prior neuronal loss (88), and severely reduced hippocampal activity, including reductions in blood flow (89). Recently, one study comparing mild and heavy drinkers demonstrated no significant impairment of general cognition but an increased fMRI blood-oxygen-level-dependent (BOLD) response, an indicator of regional activity, in the hippocampus during correct responses to the visual encoding and memory task, implying a compensatory mechanism for cognitive function (90). However, tasks capable of identifying explicit hippocampal-sensitive cognitive impairments in adults, particularly those with substance dependency issues, are scarce beyond those investigating episodic memory. Episodic memory, or the function of remembering events in specific spatial and temporal context (in contrast to factual or semantic memory), is an important hippocampal function in humans (91, 92) and has been demonstrated to be significantly impaired in alcoholic patients (93–96). However, it should be noted that as described by Noel et al. (96), episodic memory is also sensitive to alcohol-induced damage to the PFC, so the findings of reduced episodic memory function cannot be explicitly attributed to impaired hippocampal function.

## Recovery of Cognitive Capacities

A strong body of evidence in alcohol-dependent individuals has demonstrated that various cognitive capacities do return to (or nearly to) non-dependence levels of performance. However, the details of this recovery vary widely in terms of temporal resolution based primarily on the cortical structure of interest, and it is difficult to disseminate apparent recovery of damaged regions from compensation by other cortical regions with regards to behavioral function and performance alone. For example, studies appear to suggest that cognitive deficits due to PFC damage from alcohol abuse recover on a shorter timescale compared with those dependent on the hippocampus. However, as the functionality of the PFC and hippocampus is intricately related, there is a clear challenge to designing studies to directly address the explicit temporal recovery of specific structures in humans. Therefore, the findings presented here are from studies addressing broader questions of functionality in alcoholics.

With respect to the PFC damage, recovery of cognitive function in this region is critical to the persistence of abstinence from alcoholism and avoidance of relapse in dependent individuals (97). A recent met-analysis of human literature (62 sources in all) demonstrated that cognitive impairments sensitive to the PFC in individuals with AUDs identified in recent abstainers (98–101) are primarily alleviated or “normalized” (meaning performance is comparable to non-dependent individuals) by 1-year abstinence of alcohol use (102). Similarly, improvements in executive functioning occurring as soon as 6 months into abstinence has been reported (95, 103). However, as proposed and reviewed by Oscar-Berman et al. (104), it is plausible that the recovery of PFC function is more the result of compensatory activity in associated regions of the cortex rather than distinct recovery or repair of the PFC itself.

With regard to hippocampal functionality, human studies evaluating episodic memory in dependent, long-term abstinent individuals have reported similar findings to those relating to the PFC, but the outcomes of the studies have not been entirely equivocal. For example, multiple studies have reported impaired performance on tasks of episodic memory (105–107), and that “normalization” of episodic memory performance in alcohol-dependent subjects has taken place by 1 year of abstinence (95). However, there is evidence that hippocampal dysfunction remains impaired years after abstinence (5, 108). The potential distinction of these two seemingly disparate findings may be the result of (A) many of the studies not evaluating function beyond 1-year abstinence and (B), as described previously, episodic memory is not entirely exclusive of hippocampal function. Therefore, it is possible that, while episodic memory function returns, other facets of hippocampal function remain perturbed long into abstinence from alcohol. Taken together, the current evidence suggests that the recovery of cognitive functionality in abstinent alcohol-dependent individuals is sensitive to the duration of the abstinence period, with the PFC returning to “normative” levels prior to the hippocampal formation.

## Limitations of Clinical Findings

A wealth of evidence from clinical findings demonstrates that acute alcohol exposures can inhibit cognitive capacities. Interestingly, it is primarily following withdrawal from chronic alcohol exposure that individuals experience persisting, severe cognitive impairments. As eloquently described in Oscar-Berman et al. (104), studies involving human subjects and drugs of abuse are often rife with complicating and confounding factors, including family history, genetic predisposition, and past life events and experience, much of which cannot be controlled for. While clinical studies are limited to observational investigations into the deleterious cortical adaptations subsequent to chronic alcohol exposure, preclinical models have been successful at informing and elaborating our understanding of the cellular and molecular changes, which may explain the mechanisms underlying cognitive disparities in abstinent alcohol-dependent subjects. Further, preclinical models of alcohol dependence have generated evidence suggesting that the distinct cellular compositions of the PFC and the hippocampus may be the basis for the differential cognitive recovery in these regions in abstinent individuals. Therefore, the following sections will discuss preclinical models of alcohol addiction and dependence with specific focus on cognitive impairments dependent on the PFC and hippocampus and will elucidate the associated cellular and molecular changes in these regions.

## Impact of Alcohol on Cognition: Preclinical Findings

Rodent models of alcohol dependence have been instrumental in furthering our understanding of both the cognitive and neurobiological impact of withdrawal from alcohol dependence, as well as providing critical insight into the potential mechanisms of the pathological state associated with and resulting from alcohol withdrawal in dependent animals. While studies targeting examination of one explicit region or feature are impossible in human populations, particularly with regards to the effects of drugs of abuse, animal models have been instrumental tools in allowing for the fine manipulation of explicit cortical regions and functions.

## Alcohol Impairs PFC Function

Multiple studies employing rodent models have investigated the impact of alcohol dependence on prefrontal cognitive capacity. Growing evidence suggests that the rodent medial prefrontal cortex (mPFC) likely represents a functional homolog of the human medial and dorsolateral PFC (109). Reports using various rodent models of alcohol dependence [including chronic intermittent ethanol vapor exposure (CIE), liquid diet, two bottle choice; for paradigm overviews, see Ref. (110)] have found behavioral inflexibility (111), impaired extinction (112), impaired set-shifting (113), and impaired working memory (114, 115), all tasks which require a fully functioning PFC. Further, two of these studies (112, 113) linked the disruption in frontal cortical function to alcohol-induced dysregulation of the *N*-methyl-d-aspartate glutamatergic receptor (GluN) system. Two studies have investigated PFC functions into periods of abstinence following chronic ethanol exposure via CIE (10 days abstinence; (116)), or liquid diet (6 weeks abstinence; (114)). Interestingly, at 10 days into abstinence there is a lack of impairment in cognitive flexibility while at 6 weeks into abstinence there were severe impairments in working memory. Furthermore, investigation of anxiety-like behavior, 6 weeks into abstinence, demonstrated a lack of emotional behavioral deficit in abstinent animals (114). Taken together, it is evident that the paradigm of ethanol experience and the type of behavioral investigation are critical when determining alterations in PFC-dependent functions during abstinence, and that some PFC-dependent behaviors are less sensitive to the neurobiological alterations in the PFC in abstinent animals compared with others.

## Alcohol Impairs Hippocampal Function

Animal models have also been critical in resolving the explicit impact of chronic alcohol on the functionality of the hippocampus. Similar to the studies in animal models of alcohol dependence, which replicated the PFC impairments observed in humans, studies in animals exposed to translationally relevant models of chronic alcohol exposure have reproduced and expanded on the findings from human subjects. These studies have resulted in numerous structural and functional abnormalities of the rodent hippocampus similar to those seen in human studies. For example, studies in rodents employing forced chronic consumption demonstrate long-term exposures to alcohol resulted in extensive impairment in spatial memory (117–122). Unfortunately, behavioral disparities in these preclinical models have been limited to the spatial and contextual processing functions of the hippocampus with no reference to the temporal discrimination role of this structure. Nevertheless, it is clear that chronic alcohol exposure critically impairs hippocampal function in preclinical models similar to those previously discussed in clinical settings, although there remain unanswered questions in this field with regard to the complete profile of hippocampal cognitive impairments. The remainder of the review will focus on the hippocampus and provide a brief overview of the cellular and molecular mechanisms in the hippocampus that could contribute to the long-term impairments in the behaviors dependent on the hippocampus in preclinical models of AUDs.

## Molecular Actions of Alcohol in the Hippocampus

### Acute Effects on GluNs

**Figure 1:**
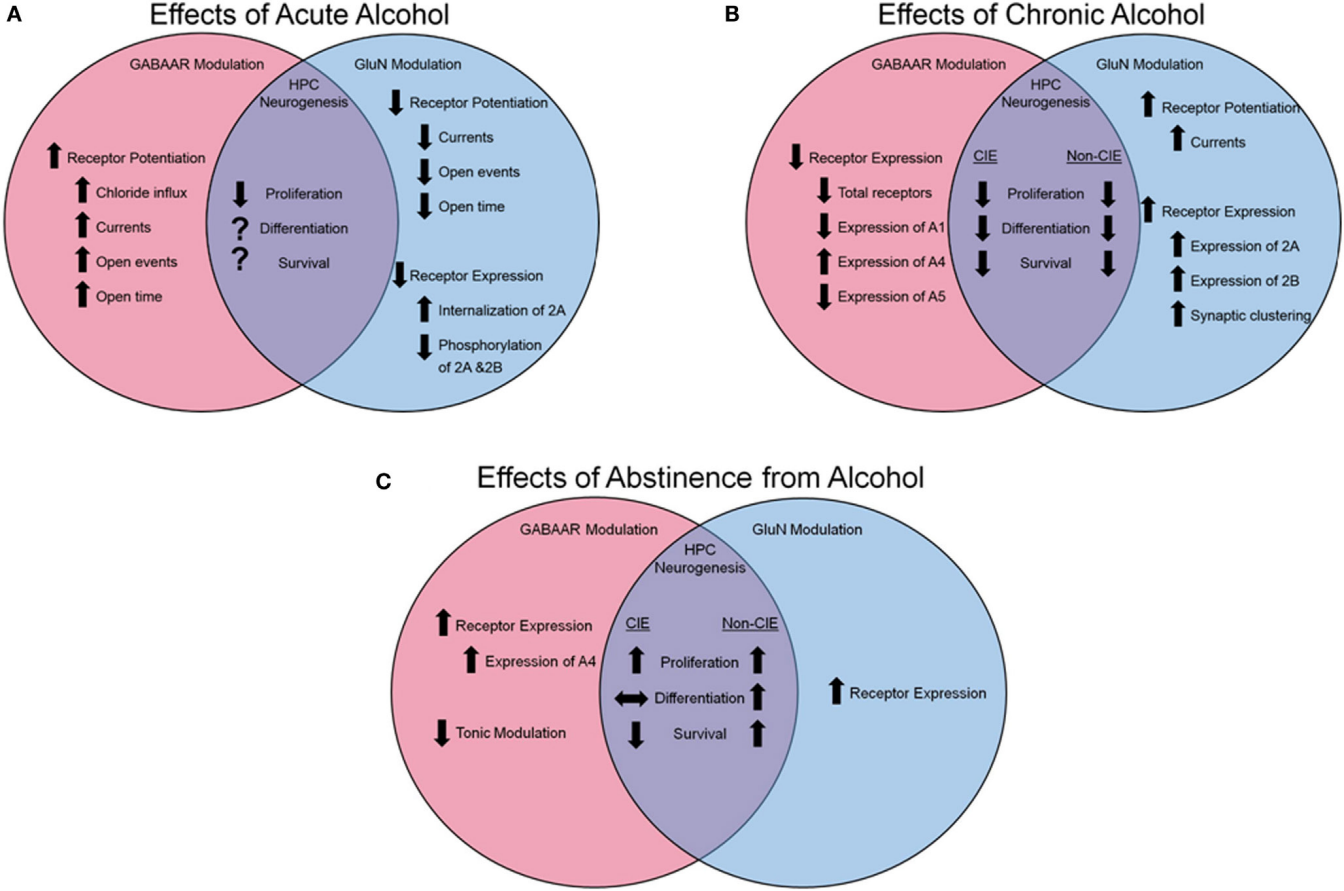
**Effects of alcohol on GABAa and GluN receptor modulation and hippocampal neurogenesis**. **(A)** Influence of acute alcohol exposure on receptor function and expression and HPC neurogenesis. **(B)** Influence of chronic alcohol exposure on receptor function and expression and HPC neurogenesis. **(C)** Influence of abstinence from alcohol on receptor function and expression and HPC neurogenesis. Arrows pointing up indicate an increase, arrows pointing down indicate a reduction, arrows pointing side to side indicate no change, and a question mark indicates that information is not available.

Animal models of acute alcohol exposure have been instrumental in elucidating our understanding of the molecular actions of alcohol with regard to excitatory and inhibitory transmission in the mammalian cortex (see Figure 1A for a summary). GluNs are one of the main components of excitatory transmission in the hippocampus (as well as the cortex at large) and are critical for learning and memory (123). The receptors are comprised of four subunits, two obligatory GluN1 subunits, and two additional subunits, which can be any of GluN2A-D or GluN3A-B. Evidence suggests that the 2A and 2B subunits, expressed in high density in the hippocampus, are particularly sensitive to alcohol’s inhibitory effects (124–127). Further, early evidence suggests that alcohol dose-dependently inhibits GluN-dependent current in cells (8) by decreasing the time the channel spends open (128).

### Acute Effects on Gamma-Aminobutyric Acid A Receptors

Inhibitory transmission plays a similarly critical role in cognition, learning, and memory in the hippocampus (and the cortex at large) (129). In addition to alcohol’s reduction of glutamatergic transmission *via* impairment of GluN function, alcohol also acts as a non-competitive agonist, directly enhancing the chloride transmission of the gamma-aminobutyric acid A (GABAa) channel (130) effectively hyperpolarizing the neural cells (see Figure 1A for a summary). Similar to GluN, the GABAa receptor (GABAaR) is comprised of five subunits, typically two alpha (A1-6), two beta (B1-3), and one subunit, which could be comprised of a gamma (G1-3) or delta. However, unlike GluN, the precise site of action on a given subunit is of debate [reviewed in Ref. (21)], with many subunits demonstrating sensitivity to alcohol (131), and much evidence is contradictory; for example, Wallner et al. (20) suggested that the B3 subunit was mediating the receptor’s sensitivity to alcohol, but this was later contradicted in a mutant mouse model void of the B3 subunit, but still demonstrated GABA-ergic enhancement following alcohol administration (132). It is highly possible that alcohol’s capacity to enhance inhibitory function of the GABAaR is dependent on the specific conformation of subunits instead of acting at a single subunit.

### Chronic Effects on GluNs

*N*-methyl-d-aspartate glutamatergic receptors and associated intracellular signaling molecules adapt to the reoccurring presence of alcohol, facilitating the development of the dependent phenotype. Post-translationally, the GluN2B subunit is phosphorylated subsequent to alcohol exposure (133), particularly in the hippocampus (13), resulting in an increase in receptor function. Over repeated alcohol exposures, an increase in expression of GluN subunits 2A and 2B (134, 135), synaptic-specific clustering of GluNs (136), as well as an increase in GluN-mediated currents (136) are observed (Figure 1B). It is probable that this increase in expression and function of the GluN receptor is a compensatory mechanism against chronic alcohol’s impairment on the receptor; however, when alcohol is absent from the cortical system during withdrawal, the pathologic over-expression of GluNs (137), along with the normalized GABA-ergic function in the absence of alcohol’s facilitating effects, results in cortical hyperactivity and excitotoxicity.

### Chronic Effects on GABAaRs

In addition to the molecular changes observed in the GluN system following long-term alcohol exposures, the GABAaRs are subject to dynamic regulation by the drug (see Figure 1B for a summary). The subunits of the GABAaR are differentially expressed subsequent to chronic alcohol in a region- and subunit-specific manner [for detailed review see Ref. (138)]. Evidence suggests an exchange of subunits expressed on the cell surface with a reported reduction in A1 subunits in the hippocampus (139) and an increase of A4 (140–142) and A5 (139) following CIE. However, subunit expression is not the only element of GABAaR modulation that is altered by chronic alcohol exposure. Following withdrawal from CIE, neurons displayed heightened excitability, which was pharmacologically attributable to increases in the number of A4 containing GABAaRs (142) as well as reductions in tonic current modulators (143), increase in A4 synaptic localization (144), and subunit-specific changes in trafficking (145), leading to a preferential increase in A4 expression over other subunits. Therefore, following chronic alcohol exposure, there is a generalized reduction of GABAaR functionality, leading to heightened neuronal activity in the absence of alcohol’s modulating effects.

## Potential Biological Mechanism of Hippocampal Sensitivity to AUDs: Impact of Altered GluN and GABAR Signaling in the Hippocampus on Adult Neurogenesis

The regionally differential rates of cognitive recovery following abstinence from alcohol use are potentially consequent to the neurogenic properties (or lack thereof) of each region. To be more specific, cognitive function relying on the frontal cortical region in humans has been described as being recovered at an earlier time in abstinence than cognitive functions specific to the hippocampal formation of the limbic system as previously discussed. It is possible that this disparity is due to, at least in part, the ongoing adult neurogenesis in the hippocampus which occurs at a much lesser rate in the PFC of mammals (146); neurons which would be generated during critical periods of withdrawal would be developing into mature neurons during a time of negative affect (147, 148), potentially resulting in a pathologic phenotype and dysfunctional characteristics (149). This problematic phenomenon would be far more impactful in a region with high neurogenesis (such as the hippocampus) as compared with a region of low or absent neurogenesis, where the typical functioning of the existing circuitry may return upon complete washout of the drug.

Adult mammalian neurogenesis is a widely accepted phenomenon, as evidence demonstrates the existence of mitotically active cells in distinct regions of the brain, one which is the granule cell layer of the DG of the hippocampus. Neurogenesis, or the process of proliferation, differentiation, and maturation of neural progenitor cells to fully functional and integrated neuronal components of the surrounding network (150, 151), has been confirmed in numerous mammalian species, including humans (152). Assessment of cell number and structure at various time points following cell birth can provide insight into the impact of exogenous factors on the neurogenic process in the hippocampus [for comprehensive review of granule cell development see Ref. (153)].

The explicit functionality of these adult-born cells is still a topic of contention. Hippocampal-sensitive learning has been shown to positively influence proliferation and survival of new neurons [reviewed in Ref. (154)]; inversely, increases in proliferation or survival of newly born neurons can increase performance on hippocampal-sensitive tasks, while reductions or ablations of neuronal proliferation results in problematic cognitive performance [reviewed in Ref. (155)]. Acquisition, retention, and extinction of trace fear conditioning (TFC; a hippocampus sensitive task) has been shown to be sensitive to changes in neurogenesis (156) due to or as a result of its hippocampal-dependence (157), but as yet, investigations into the impact of clinically relevant models of chronic alcohol on TFC performance have not been reported.

### Regulation of Neurogenesis by GluNs

Glutamatergic signaling *via* GluNs is of critical importance in regulating neural stem cells in the hippocampus, particularly in the withdrawal/abstinence period in alcohol-dependent subjects. Under basal conditions, some stages of immature neural progenitors (proliferating and differentiating cells) in the hippocampus express GluNs (158). When coupled with the evidence that GluN-dependent long-term potentiation in the DG can increase progenitor proliferation (159, 160) and survival (159), these findings imply that regulation of hippocampal neurogenesis is sensitive to GluN stimulation on newly born granule cells. Alcohol’s long-term actions *via* GluNs would, therefore, affect proliferation, survival, and function of the newly born neurons in a dynamic manner which would change over the course of abstinence from alcohol. Alcohol, as described previously, has the consequence of maintaining GluNs at the synapse, effectively impairing cycling of receptors back into the cell for degradation or reuse. Therefore, the role of alcohol on hippocampal neurogenesis would be mediated by either GluN dysregulation, GABA-ergic dysregulation, or a balance of both.

### Regulation of Neurogenesis by GABAaRs

The granule cells of the hippocampus are maintained in a quiescent state by the mossy fibers of the hilus *via* GABA-ergic regulation [reviewed in Ref. (161)]. Evidence has demonstrated that these cells do express GABAaRs (162), as do the surrounding cells of the DG (163, 164); therefore, not only are the granule cells sensitive to enhanced GABA-ergic transmission during exposure to chronic alcohol but are also subject to secondary regulation due to the modulation of activity of surrounding cells by alcohol’s actions on the GABAaR. As specific subunit compositions of the GABAaR can modulate important stages of neurogenesis (particularly the maintenance of quiescent cells and proliferation), this could provide a potential mechanism by which alcohol could be modulating neurogenesis in dependent individuals. During periods of alcohol intake, GABAaR function would be supported and facilitated such that quiescent cells would be maintained (165, 166) as such and proliferation would be reduced (167–169). In the acute absence of alcohol, the facilitation of GABAaR activity would be lost and quiescent cells would be allowed to proliferate, and these effects could result in increase or decrease in cell survival in the days following withdrawal (169–171). However, impaired GABA-ergic receptor function has been shown to restrict morphology of newly born cells (172), which could reduce the number of synaptic connections and network integration required for survival and function of the granule cells and, therefore, result in net reduction of the number of surviving cells during protracted abstinence (171). This finding serves as a potential argument for the reduced survival subsequent to the increased proliferation following withdrawal in dependent animals (171).

### Regulation of Neurogenesis by Alcohol

In addition to a general understanding of neurogenesis, we are beginning to understand how alcohol exposure impacts hippocampal neurogenesis and what this may imply for cognitive performance and capacity (see Figures 1A–C for a summary). For example, while cellular proliferation and neurogenesis are reduced during excessive alcohol-induced dependence (167–169), early withdrawal from excessive alcohol is documented to result in an increase in cellular proliferation in the DG (169–171). The survival capacity of progenitors born during this period of increased proliferation and their functional importance is still unclear; however, reports using alcohol gavage [blood alcohol levels (BALs) reaching >400 mg%] demonstrate increased survival of newly born neurons subsequent to the proliferative burst (170, 173, 174). In contrast, animals made dependent to alcohol *via* ethanol vapor exposure (BALs maintained between 150–250 mg%) demonstrate a marked reduction in the number of surviving young neurons in the DG (169, 171). This difference could be attributed to differences in BALs and negative affect symptoms resulting from the exposure paradigm (gavage vs. CIE). Unfortunately, there is no conclusive evidence linking aberrant neurogenesis subsequent to alcohol dependence and impaired hippocampal cognitive function. Future studies will be required to demonstrate the plausibility of this mechanism as an underlying explanation for the deleterious effect of alcohol dependence on hippocampal function.

## Summary and Conclusion

The goal of this review was to provide initial evidence in support of the proposal that the cognitive recovery of the hippocampus and the PFC following abstinence from long-term alcohol abuse occur at different rates, potentially due to their difference in cellular composition and neurogenic functionality. For example, clinical evidence supports recovery of certain PFC-dependent tasks in times of abstinence from alcohol at different rates compared with hippocampal-dependent tasks. Preclinical findings in animal models of alcohol exposure support the clinical observation; mechanistic studies support that this temporally differential rescue of PFC-dependent tasks is potentially due to the neurogenic deficits in the hippocampus during abstinence, such that the birth of new neurons during periods of negative affect result in the persistence of the hippocampal-specific cognitive disparities.

## Future Perspective

Many questions remain unanswered with regard to human hippocampal function during periods of alcohol abstinence. For example, it is clear that employing cognitive therapy can support individuals in successful attempts at abstinence. Given that extinction training is being adopted in clinical behavioral therapy to promote recovery from relapse (175), it is critical to investigate similar potential therapeutic strategies (be it behavioral or pharmacological), which will serve this purpose not only to ameliorate the cognitive disparities in these individuals but to facilitate dependent individuals in avoiding relapse to alcohol abuse.

## Author Contributions

MS was responsible for the article concept and drafted the manuscript. CM and MS provided critical revision of the manuscript for important intellectual content. Both authors critically reviewed content and approved final version for publication.

## Conflict of Interest Statement

The authors declare that the research was conducted in the absence of any commercial or financial relationships that could be construed as a potential conflict of interest.

## Abbreviations

AUD, alcohol use disorder; BALs, blood alcohol levels; BOLD, blood-oxygen-level dependent; CIE, chronic intermittent ethanol vapor exposure; DG, dentate gyrus; fMRI, functional magnetic resonance imaging; GABAa, gamma-aminobutyric acid A subunit; GABAaR, GABAa Receptor; GluN, *N*-methyl-d-aspartate glutamatergic receptor; PFC, prefrontal cortex; TFC, trace fear conditioning.
